# Leisure activities and attitude of institutionalized elderly people: a basis for nursing practice^1^

**DOI:** 10.1590/0104-1169.3650.2556

**Published:** 2015

**Authors:** Vivian Carla de Castro, Lígia Carreira

**Affiliations:** 2 MSc, RN, Hospital Universitário de Maringá, Universidade Estadual de Maringá, Maringá, PR, Brazil; 3 PhD, Adjunct Professor, Departamento de Enfermagem, Universidade Estadual de Maringá, Maringá, PR, Brazil

**Keywords:** Health of Institutionalized Elderly, Attitude, Leisure Activities, Homes for the Aged, Geriatric Nursing, Aged

## Abstract

**Aim:**

to identify the leisure activities performed in Long-Stay Institutions for the Elderly (LSIEs), registered in the city of Maringá-PR, Brazil, and to analyze the attitude of the elderly people toward leisure promoted by the institutions.

**METHOD:**

this was a descriptive and transversal study with a quantitative approach, carried out with 97 elderly people, through the establishment of the socio-demographic profile and the application of the Leisure Attitude Scale. The data was subjected to descriptive statistical analysis, association tests (chi-square or Fisher's) and Spearman's correlation.

**RESULTS:**

males, aged 80 or over, widowed, with one to eight years of study, who had a monthly income were predominant. Age group and income were significantly associated with the performance of leisure activities. The results reflected the positive attitude of the elderly people in relation to leisure activities, except in the behavioral component.

**CONCLUSION:**

the findings of this study indicate the need for further investigation into the difficulties linked to the attitude toward leisure in the behavioral component, considering aspects such as individual concepts of leisure and the health status of the elderly people.

## Introduction

Throughout history, a vast and concrete theoretical foundation has been constructed related to leisure, from classical philosophy, gaining strength with discussions about leisure in industrial society, to the current contributions it has received from Sociology, Anthropology, Architecture and Urbanism, among others^(^ 1 ^)^. In this study, leisure can be understood as an occupation for which the individual can give himself freely, whether for relaxation, for fun, recreation and entertainment, or to develop their free formation, their social participation or their creative ability, after freeing themselves from their professional, family and social obligations^(^ 2 ^)^.

One of the determinant factors for involvement in leisure activities is the attitude toward leisure, with a positive attitude tending to be related to greater involvement in these activities^(^ 3 ^)^. The measurement of the attitude toward leisure therefore becomes essential for structuring interventions to promote a positive attitude and, therefore, the biopsychosocial well-being^(^ 3 ^)^.

Among the different definitions and theoretical approaches to the attitude found in the literature, there are models that operationalize the concept of attitude around the cognitive, affective, and behavioral components. The cognitive component refers to the knowledge, opinions and beliefs expressed in the attitude; the affective component refers to the feelings and physiological responses revealed in the attitude; and the behavior component is related to the structure of the behavior, preparing the individual to act in a certain way^(^ 3 ^)^.

Leisure, as well as a constitutional right, is considered a basic human need. Nursing, the essence of which is integral care for human beings, finds the support to consolidate its work process in the Theory of Basic Human Needs^(^ 4 ^)^, in which psychobiological, psychosocial and psychospiritual needs can be distinguished.

Nursing care involves all the phases of the life cycle and should be mainly centered on vulnerable groups. With aging, the risk of developing vulnerabilities increases due to the characteristic biological decline of senescence, which interacts with socio-cultural and economic aspects accumulated throughout life^(^ 5 ^)^. Regarding institutionalized elderly people, this vulnerability tends to increase.

The institutional context exhibits roles characterized by dependency, limited physical space and a determined time for daily activities, with it being vital to adapt to the rules and routines of the new environment, which does not always provide adequate living conditions, in this case, with an emphasis on non-regimented leisure as a way to exercise autonomy.

Although the classical theoretical concepts^(^ 2 ^)^ related to the theme have been marked by the Work-Leisure dichotomy, the current concepts reflect on the complementarity between one and the other. This interpretation is in agreement with the Theory of Communicative Action^(^ 6 ^)^, in which leisure is essentially a social relationship that is expressed in the "world of life", i.e., not directly related to the "world of work", through the integration between people, the pursuit of fun and the desire to feel pleasure^(^ 7 ^)^. This perspective highlights leisure activities in certain populations, in which time is not linked to labor activities, as in the case of institutionalized elderly people, who no longer perform work functions.

Given the above, this study aimed to identify the leisure activities performed in Long-Stay Institutions for the Elderly (LSIEs), registered in the municipality of Maringá-Paraná, Brazil, and to analyze the attitude of the elderly people toward leisure promoted by the LSIEs.

## Methodology

The population of this cross-sectional descriptive and quantitative study was composed of 297 elderly people, with similar proportions regarding gender (148 men and 149 women), residents of seven Long-Stay Institutions registered with the Department of Social Welfare and Citizenship (CASS) of the municipality of Maringá-PR. The study included participants aged 60 or over, who had resided for at least six months in the LSIEs of Maringá-PR and were able to respond to the study questions, indicated through obtaining the minimum score in the Mini Mental State Examination (MMSE) cognitive evaluation test^(^ 8 ^)^. The study included 97 elderly people.

Data collection took place between January and March 2013, through the application of an instrument for the establishment of the socio-demographic profile of the elderly people, prepared by the researcher, and application of the Leisure Attitude Scale, for the measurement of the cognitive, affective and behavioral aspects of the attitude of the elderly people toward leisure.

Originally constructed in 1982^(^ 9 ^)^, translated into Portuguese and validated in 2006^(^ 3 ^)^, the Leisure Attitude Scale is the only scale that measures the three components of attitude separately. It consists of 36 items divided into three subscales and uses a Likert type scale of five levels as the response system. The values of the measures of attitude are obtained by arithmetic addition of the responses given by the participants for the respective items. For each subscale the total minimum value is 12 points and the maximum 60 points, with the neutral point situated at 36. Thus, the total scale has a minimum value of 36 points and a maximum of 180 points with the neutral point situated at 108. Values above the neutral point reveal a more positive attitude, while those below the neutral point indicate a more negative attitude toward leisure.

The cognitive attitude is related to the assertions of leisure as a good way to use the time, a benefit for individuals and societies, the possibility of creating friendships, and the contribution to health, productivity at work and self-growth. Regarding the affective attitude, statements are scored on pleasure, good experiences and the revitalization provided by the leisure, as well as its value as a way to pass the time or a possibility for self-expression. With regard to the behavioral attitude, this includes assertions about the planning, opportunities and resources for the frequent performance of leisure activities^(^ 3 ^)^.

The data for the characterization of the subjects concerning institutionalization and leisure, as well as the measurement of positive and negative attitudes toward leisure, were analyzed descriptively. Leisure activities mentioned by the elderly people were classified according to the following categories: physical, manual, artistic, intellectual, associative and touristic^(^ 10 ^)^. The association between the categorical variables and the performance or non-performance of leisure activities was verified through the chi-square test or Fisher's exact test. The correlation between the subscales that comprise the Leisure Attitude Scale, including the total scale, was verified by calculating Spearman's correlation coefficient. The Statistical Analysis System (SAS) 91.1 software was used to perform the statistical tests. The significance level for the statistical tests was 5%.

The study was approved by the Human Research Ethics Committee of the State University of Maringa (COPEP/UEM) under authorization No. 160.445, in accordance with Resolution 466/12. The participants agreed to participate in the study by signing the Informed Consent (IC) form.

## Results

Of the 97 institutionalized elderly study participants, 55 were male (56.7%) and 42 female (43.3%). Elderly people aged 80 and over (41.2%), widowed (38.5%), who had from one to eight years of formal education (46.4%) and had some monthly income (86.6%) were predominant.

Regarding the institutions, four were private and three philanthropic and/or governmental, however, 64% of the study sample were living in the latter type. The length of residence in the LSIE was one to five years for 49.5% of the elderly people. The main reason to seek accommodation in these places was due to difficulty in maintaining the family relationship (66%), followed by self-care difficulties (16.5%), financial deficit (12.4%) and due to their own desire (5.1%).

Concerning leisure activities, 69% of the elderly people reported performing them, while the remaining 31% reported that their respective LSIEs did not offer leisure activities, or that they were not suitable for their health conditions, or even that they did not felt the desire to perform them. The classification of the activities performed by the elderly people, as well as their frequency and duration, are listed in Table 1.

**Table 1 -:** Characteristics of the activities performed by institutionalized elderly people with access to leisure (n=67). Maringá, PR, Brazil, 2013

Leisure	Categories	n	%
Activities	Physical	13	19.4
	Manual	20	29.9
	Artistic	10	14.9
	Intellectual	39	58.2
	Associative	30	44.8
	Touristic	07	10.4
Frequency	1 time per week	10	14.9
	2 times per week	12	17.9
	3 times per week or +	45	67.2
Duration	Up to 1 hour	19	28.4
	1 to 2 hours	17	25.4
	≥ 2 hours	31	46.2

Physical activities included walking, stretching and gymnastics performed in the gymnasium for senior citizens (GSC). Manual activities referred to plant cultivation and craftwork, as well as the optional assistance in the LSIE services. Artistic activities included drawing, painting, origami, music, parties and cultural dance and theater performances. The intellectual activities were linked to reading, a literacy program, television and radio. Associative activities included conversations, games (cards, dominoes, bocce and bingo) and religious occasions, and the touristic activities were associated with trips performed under the direction of the LSIE, for example, fishing, parks and shopping centers.

It is noteworthy that almost 60% of the subjects who performed leisure activities practiced intellectual activities and more than half of these (51.2%) reported watching television, while 7.7% mentioned reading as a spontaneous and enjoyable way to use the time. In relation to the associative and manual leisure activities, the second (44.8%) and third (29.9%) categories, respectively, most mentioned by the elderly people who practiced leisure activities, games (56.6%) and craftwork (50%) were emphasized, especially crochet and embroidery.

Physical activities were present in the leisure of almost 20% of the elderly practitioners, followed by artistic (14.9%) and touristic (10.4%) activities. Although not the most evident in the leisure of the elderly, physical activities were the subject of the greatest number of suggestions to complement the leisure in the LSIEs, especially with regard to the practice of sports (football, swimming and table tennis), dance and hydrogymnastics. In addition to these, the possibility of living with pets and children was also suggested.

When associating the performance of leisure activities with the sociodemographic and institutionalization characteristics, statistically significant differences were observed regarding age group (p <0.010) and income (p <0.04), as shown in Table 2. It was found that 40.3% of the subjects who performed leisure activities were in the 60 to 69 years age group, while among those who did not perform leisure activities, 63.3% were 80 years or over.

**Table 2 -:** Access of elderly people to leisure, according to the sociodemographic and institutionalization characteristics. Maringá, PR, Brazil, 2013

Variables	Categories	Leisure					Total	p-value
		Yes			No			
		n	%		n	%	N	
Gender	Male	40	59.70		15	50.00	55	0.3728
	Female	27	40.30		15	50.00	42	
Age Group	60 to 69 years	27	40.30		05	16.70	32	0.010*
	70 to 79 years	19	28.40		06	20.00	25	
	80 years or +	21	31.30		19	63.30	40	
Marital Status	Single	25	37.30		10	33.30	35	0.5219
	Separated	18	26.90		06	20.00	24	
	Widowed	24	35.80		14	46.70	38	
Income	Do not have	12	17.90		01	03.30	13	0.040^†^
	Have	55	82.10		29	96.70	84	
Education	Illiterate	22	32.80		10	33.30	32	0.9900
	1 to 8 years	40	59.70		18	60.00	58	
	≥ 8 years	05	07.50		02	06.70	07	
Length of Institutionalization	6 months to 1 year	13	19.40		09	30.00	22	0.4936
	1 to 5 years	34	50.75		14	46.70	48	
	≥ 5 years	20	29.85		07	23.30	27	
Character of the LSIE	Government/Philanthropic	46	68.70		16	53.30	62	0.1463
	Private	21	31.30		14	46.70	35	

* Chi-square test, p-value<0.05

† Fisher's exact test, p-value<0.05

The results regarding the Leisure Attitude Scale reflect, in general terms, the positive attitude of the elderly people in relation to leisure activities, except in the behavioral subscale, the mean (35.8) of which was below the neutral point 36. The cognitive subscale had the highest mean (54.9) compared with the others, revealing the more positive attitude of the elderly people toward leisure in this component.

The cognitive and affective subscales presented a positive attitude toward leisure in 96.9% of the elderly people, showing the same pattern of behavior (positive) for both. In the behavioral subscale, however, although there was a slight positive trend (51.5%), it was clear that there were two groups with different patterns of behavior, that is, they presented a positive attitude and a negative attitude toward leisure.

The minimum values demonstrated the existence of responses that indicated a negative attitude in all sub-scales and the total scale, even though the minimum possible value was not reached, unlike the maximum value, achieved in two of the subscales (Table 3).

**Table 3 -:** Descriptive statistics of the attitude toward leisure of institutionalized elderly people. Maringá, PR, Brazil, 2013

Subscale	No. of items	Mean	SD*	Min^†^	Max^‡^	Positive^§^			Negative^||^	
						n	%		n	%
Cognitive	12	54.90	5.95	32	60	94	96.90		3	3.10
Affective	12	53.50	6.88	23	60	94	96.90		3	3.10
Behavioral	12	35.80	8.16	14	54	50	51.50		47	48.50
Total	36	144.20	17.64	83	168	93	95.90		4	4.10

*Standard Deviation

† Minimum value for each subscale and the total scale

‡ Maximum value for each subscale and the total scale

§ Positive attitude toward leisure for each subscale and the total scale

|| Negative attitude toward leisure for each subscale and the total scale

The correlations obtained between the cognitive, affective and behavioral subscales, as well as the total scale are presented in Table 4.

**Table 4 -:** Correlation test between the subscales of the leisure attitude of institutionalized elderly people. Maringá, PR, Brazil, 2013

Subscale	Affective	p-value	Behavioral	p-value	Total	p-value
Cognitive	0.552*	<0.01	0.442*	<0.01	0.702*	<0.01
Affective	1		0.559*	<0.01	0.829*	<0.01
Behavioral			1		0.873*	<0.01

* Spearman's correlation, p<0.01

It was verified that the correlation coefficient between the behavioral and affective subscales (r=0.559; p<0.01) was greater than that obtained for the behavioral and cognitive subscale (r=0.442; p<0.01) and the affective and cognitive subscale (r=0.552; p<0.01). Regarding the total scale, the correlation values ranged between 0.702 and 0.873.

## Discussion

Being female, of advanced age, widowed, with lower levels of education and income are predictors for institutionalization^(^ 11 ^)^. This draws attention to the predominance of women in the characterization of the institutionalized community, as demonstrated by a study conducted in Korea^(^ 12 ^)^. The results of the present study regarding gender, however, differ from those highlighted, since 56.7% of the elderly participants were male. It should be noted that, despite female longevity, living longer means greater exposure to adversity - discrimination in access to education, income, food, meaningful work and politics - which, taken together, lead to greater frailty in older age^(^ 13 ^)^.

Leisure activities contribute to the biopsychosocial balance of the elderly person^(^ 14 ^)^. Regarding leisure activities performed by elderly people, the findings of this study confirm those of a study conducted in a LSIE in Curitiba^(^ 15 ^)^, in which the most frequent activities were those of the intellectual, associative and manual types. These activities seem to have a protective effect on functional loss in elderly people, through mechanisms similar to the work activity, which involves cognitive stimulation and compensatory mechanisms of the social support network^(^ 14 ^)^. Simultaneously, given the importance of physical activity in preventing falls and functional disability in elderly people, especially with strength training, muscular endurance, flexibility and balance exercises^(^ 16 ^)^, the need to implement physical activity during the leisure time of institutionalized elderly people is stressed, as the elderly people themselves suggested.

The participation of the elderly people in leisure activities decreased significantly with respect to age, this reduction may be related to functional limitations, since functional performance tends to decrease with increasing age^(^ 17 ^)^. Regarding income, the association with the performance of leisure activities can be related to the grouping of many of the elderly people in the same category. However, the health, education and leisure conditions afforded by the income earned throughout life can determine the participation of the elderly people in current activities, since these conditions may have influenced the individual concept of leisure, based on the intensity of the life experiences.

Work is the main means of obtaining financial resources throughout life, therefore this also ultimately determines the time that is available for leisure. The inexistence of work in the lives of the elderly people in the institutional context makes it possible to think of leisure as part of the world of life and not linked to the world of the system, as proposed by the theoretical perspective addressed here^(^ 6 ^)^. Thus, in addition to its relaxation function, leisure can be interpreted as an instrument of empowerment of the person that encourages the pursuit of full pleasure.

The attitude toward leisure of the institutionalized elderly people was shown to be generally positive. However, when analyzing the subscales of the components of attitude separately, a negative attitude was verified in relation to the behavioral subscale. This result differs from that found in both the validation study of the original version^(^ 9 ^)^ and that of the Portuguese version^(^ 3 ^)^ of the Leisure Attitude Scale, in which the means of the responses for the three components reflected the positive attitude of the participants in relation to the leisure activities. In the present study, the cognitive subscale presented the highest mean, while the behavioral subscale presented the lowest, which corroborates the findings of the validation study of the Portuguese version^(^ 3 ^)^, however, diverges from the results of the original version study^(^ 9 ^)^, in which the highest mean was obtained in the affective subscale and the lowest in the cognitive subscale.

In the area of social psychology, contrary to what would be considered common sense, the attitude is not constituted by the action itself, but developed according to the object in question and the position of the individual regarding this, based on his/her life experiences^(^ 18 ^)^. The fact that the cognitive and affective attitudes of institutionalized elderly people toward leisure were shown to be positive may be linked to past experiences, in which the elderly people constructed beliefs and nurtured emotions related to leisure. Conversely, the behavioral attitude toward leisure divided the opinion of the elderly people regarding the presence of leisure in the day-to-day of the institutional reality, being presented negatively from the quantitative data analysis.

Individuals can change their attitude over time, as they assimilate new knowledge and have new experiences. The aging process, during which the body undergoes morphological, functional and biochemical changes^(^ 19 ^)^, can influence transformations in knowledge and beliefs, as well as in behavior with respect to an object, in this case leisure. Furthermore, behavior can be influenced by the circumstances experienced and, when considering an institutional context to which the elderly people had or have to adapt, the sudden emergence of rigid routines, the reduced privacy and the imposition of roles characterized by dependence must be considered^(^ 20 ^)^.

Regarding the correlations obtained between the subscales and the total scale, as in the original scale and the Portuguese version, the affective and behavioral subscales presented coefficients greater than those obtained in the cognitive and behavioral subscales. The discussion, in this sense, is centered on the hypothesis that behavioral intentions are determined more by what is felt than by what is known about leisure activities^(^ 3 ^)^.

The satisfaction of the basic needs of the elderly people depends on the professionals of the LSIE, especially the nursing staff, directly responsible for the provision of care. Accordingly, it should be mentioned that the nurse, as well as the team, must view the human being from the perspective of integrality, offering personalized and humanized care^(^ 4 ^)^, which includes greater attention to leisure activities.

The institutional context is subject to social interaction and empowerment of the person^(^ 6 ^)^, provided that the interventions performed consider people's choices about their lives, especially regarding leisure, which has proved relevant for the development of health promotion strategies that favor quality of life^(^ 21 ^)^.

## Conclusion

The leisure activities performed by the elderly people were mostly intellectual, followed by associative and manual. There was a statistically significant association between the performance of leisure activities and the age group and income variables. In general, the results reflected the positive attitude of the elderly people in relation to leisure activities, however, the analysis of the attitude components separately demonstrated a positive attitude toward the cognitive and affective components and a negative attitude regarding the behavioral component, which relates to the practice of leisure in the day-to-day institutional reality.

Given the confrontation between the expectations related to institutionalization and the need to adapt to new routines, leisure emerges as instrument for the empowerment of the elderly person, not only because it contributes to their biopsychosocial balance, but also because it is reflected as the freedom of choice in the pursuit of pleasure. Therefore, the findings of this study indicate the need for further investigation into the difficulties linked to the attitude towards leisure in the behavioral component, considering aspects such as the individual concepts of leisure and the health status of the elderly people, the main limitations of this study.
